# Research on Techniques of Multifeatures Extraction for Tongue Image and Its Application in Retrieval

**DOI:** 10.1155/2017/8064743

**Published:** 2017-03-30

**Authors:** Liyan Chen, Beizhan Wang, Zhihong Zhang, Fan Lin, Yihan Ma

**Affiliations:** Software School of Xiamen University, Xiamen 361005, China

## Abstract

Tongue diagnosis is one of the important methods in the Chinese traditional medicine. Doctors can judge the disease's situation by observing patient's tongue color and texture. This paper presents a novel approach to extract color and texture features of tongue images. First, we use improved GLA (Generalized Lloyd Algorithm) to extract the main color of tongue image. Considering that the color feature cannot fully express tongue image information, the paper analyzes tongue edge's texture features and proposes an algorithm to extract them. Then, we integrate the two features in retrieval by different weight. Experimental results show that the proposed method can improve the detection rate of lesion in tongue image relative to single feature retrieval.

## 1. Introduction

Chinese traditional medicine is the experience of the Chinese people during thousand years of struggle with the disease. Its curative effect is significant, and side effects are small. Compared with modern medicine, it has certain advantages and potential in healthcare, health, rehabilitation, and so forth. In the Chinese traditional medicine, there are four kinds of diagnosis methods including inspection, olfaction, interrogation, and palpation. Tongue image is an important part of inspection which gets the disease's situation by observing patient's tongue color and edge shape changes [1].

Tongue diagnosis is one of the important topics in the field of medicine at present; with the continuous deepening of the Chinese traditional medicine tongue diagnosis objectiveness research, digital images of tongue diagnosis have also been applied in the clinical work. A lot of tongue images are generated in clinical works every day, and how to retrieve and manage the increasingly large tongue database to support tongue diagnosis features extraction has become a very challenging subject. Traditional tongue images management describes the tongue image information by manual labeling and retrieves tongue image by the description information, but this way has been unable to meet the needs of large-scale tongue image database retrieval. On the other hand, traditional tongue diagnosis depends highly on clinicians' experience and thus different clinicians are likely to reach remarkably different diagnostic results for the same patient. So this paper proposes a novel method applying content-based image retrieval technology to tongue image retrieval.

In recent years, some computer image processing technologies have been used in tongue diagnosis in the Chinese traditional medicine. These methods can be divided into two categories according to different tongue image features used: color-based approaches and texture-based ones. For the former, [2–7] used the color features to analyze the tongue images. The color matching of tongue images in different color spaces with different metrics was investigated and reported in [2, 4] that proposed a method based on region partition and feature matching for color recognition of tongue images. Li and Yuen (2002) addressed the problem of color image matching in medical diagnosis. They proposed the sorted metric in coordinate space. To improve the matching performance, a probabilistic combined metric is proposed based on the theory of combining classifier. Wang et al. (2004) proposed a new tongue color calibration scheme and utilized a gradient vector flow (GVF) snake based model integrating the chromatic information of the tongue image used to extract the tongue body. Li and Liu (2009) developed a push broom hyperspectral tongue imager and discussed its spectral response calibration method. A new approach to analyze tongue color based on spectra with spectral angle mapper is presented. This new color analysis approach is superior to the traditional method especially in achieving meaningful areas of substances and coatings of tongue. Papers [8, 9] used a variety of tongue image features (such as color, texture, or shape) to match and identify tongue images. Chiu (2000) built a computerized tongue examination system (CTES) based on chromatic and textural algorithm. The chromatic algorithm is developed to identify the colors of the tongue and the thickness of its coating. Guo (2008) proposed a new color texture operator, Primary Difference Signal Local Binary Pattern. The matching performance is evaluated on color, grayscale and color texture, and fusion of color and texture features.

For these methods, each of them has its fair share of success, but corresponding limitations also accompany with them. Taken as a whole, they fail to satisfy the demands for both accuracy and robustness simultaneously, which are the basic requirements for a successful extraction. In fact, the Chinese traditional medicine attaches great importance to correlation analysis; namely, different tongues reflect different diseases, and there may exist some symbiosis or mutual exclusivity among different characteristics of the tongue. It is necessary for us to integrate a variety of tongue image features for tongue image analysis. Based on this, the paper proposes a method combining color and texture features in retrieval by different weight to improve the recognition rate for tongue diagnosis in the Chinese traditional medicine. First, we use the iterative method to extract the initial main colors and the number of them and then get the main color histogram by GLA algorithm. Considering the deficiency of expressing tongue image information by using color feature only, the paper analyzes tongue image further and puts an algorithm to get tongue image's texture feature. The new method combines the improved main color histogram descriptor and edge histogram descriptor to give different weights to the comprehensive retrieval.

To evaluate the performance of the proposed algorithm, in the course of the experiment, we used 268 tongue images as experimental samples; these images are divided into several colors in advance by the doctor.

From experimental results, we can see that the improved main color histogram algorithm is better than the traditional main color histogram in the search results. Although differences of the statistical results are small, we can obviously feel the effect of the retrieval greatly improved. At the same time, the position of the relevant tongue is also more forward and focused.

Experiment used the same tongue images ditto, which are divided into 5 texture categories. Experimental results show that improved edge histogram's retrieval precision and recall ratio is slightly higher than that of the traditional edge histogram.

Finally, in order to analyze the function of comprehensive color and texture feature in tongue image retrieval, we select a set of tongue images which have prominent color and texture features for the experiment and then randomly select a tongue image as the query image. In order to achieve better retrieval results, the weight of color and texture features is set to 0.6 and 0.4, respectively. We can see that the result is more accurate than the single feature search results. A large number of tongue image retrieval experiments show that, due to the great difference of the tongue images, using color or texture features in retrieval would be better for some tongue images.

Experiments show that new method can improve the detection rate of lesion in tongue images.

The paper is organized as follows.  Section 2 reviews some extraction methods of tongue image features. An improved main color histogram method and improved edge histogram for tongue diagnosis in the Chinese traditional medicine are proposed in Section 3. The experimental results and analysis are shown in Section 4. Finally, some conclusions are drawn in Section 5.

## 2. Related Work

### 2.1. Main Color Descriptor

Color is the basic element of tongue image and it is one of the main features for tongue image recognition. Each tongue image has its own unique color feature, which is the basic and important feature in the image. Tongue and coating color links to the body contact, representing different lesions. So the color is one of the mainstays of tongue diagnosis in the Chinese traditional medicine and has important diagnostic value.

According to the theory of visual psychology [9], human's perception to image focuses on a few representative colors, while ignoring the secondary color details. MPEG-7 provides the main color descriptor to describe the main color information of image in arbitrary irregular region, which reflects the main color of the image. The main color descriptor is used as the color feature for image retrieval, and the basic idea is as follows [10].

To image *I*, first convert the color space to *N* dimension; then, the image color can be represented by an *N* dimension vector:

(1) $$\begin{array}{c} F=\left\{\;\left\{\;C_{i},P_{i}\;\right\}\;\right\}, \end{array}$$

where *i* ∈ [0, *N* − 1], *C*_*i*_ represents the image color after quantifying, and *P*_*i*_ represents a percentage of the corresponding quantitative color for the whole image. Sorting *F* by descending order, the traditional method is to use *P*_*i*_ ≥ 5% as the main color.

GLA algorithm is an iterative clustering algorithm searching the optimal vector quantizer for target; it is an iterative split and union process.  Algorithm 1 describes the GLA algorithm step [11].

### 2.2. Edge Histogram Descriptor

In the process of the Chinese traditional medicine, discriminating the tongue color and analyzing the lingual teeth marks, prick, crack, addiction, and other features are necessary; these features belong to the category of texture analysis. The edge texture's distribution is an important texture information and the edge histogram descriptor recommended by the MPEG-7 is widely used in the texture features of image retrieval based on the notion that, especially when the image texture distribution is not uniform, the descriptors' effect would be better when used for image matching [10].

Edge histogram describes five types of edge space distribution, containing four kinds of directional edges and a nondirectional edge. The basic idea of the algorithm is to divide the image into several subblocks and calculate the value of each subblock edge, depending on the direction of the subblock edge cumulative statistics and the edge histogram of the whole image, as follows:

1. Divide the image into 4 × 4 subimages.
2. Divide each subimage into smaller subimage blocks.
3. According to the edge detection operator defined by MPEG-7, calculate five kinds of edge values of each subimage block. If the maximum edge value is greater than a certain threshold, set the direction as the edge direction of the image block.
4. Get the 5 bin edge histogram of the subimages from the edge direction of image block and finally calculate the 80 bin histogram of the whole image.

Assuming that there are two images *Q* and *T* and their edge histograms are *H*_*q*_ and *H*_*t*_, then *Q* and *T* can use the Minkowski formula where *r* = 1 to measure the similarity as follows:

(2) $$\begin{array}{c} D\left(\;Q,T\;\right)=\overset{79}{\underset{m=0}{\sum }}\left|\;h_{q}\left[\;m\;\right]-h_{t}\left[\;m\;\right]\;\right|. \end{array}$$

## 3. Methods

The paper combines an improved MPEG-7 main color descriptor and improved edge histogram. They can overcome the problem of inaccurate retrieval by using single image feature, and they can improve the efficiency of retrieval. The two algorithms are introduced in detail in the following.

### 3.1. Improved Main Color Extract Algorithm

Each tongue image has its own unique color feature, which is the basic and important feature of the image. According to previous literature [8], there is no obvious difference between different kinds of RGB colors when using computer to automatically classify and identify tongue images in RGB color space. Therefore, RGB color space is difficult to represent the color features of different tongue images; this color space cannot classify the tongue image color. When using HSV color space to classify tongue image color, hue *H* angle value in turn increases according to the order of purple tongue, purple red tongue, pink tongue, yellow-coating tongue, pale tongue, and white-coating tongue's color feature; the saturation *S* value decreases and brightness *V* value increases in turn of purple red tongue, purple tongue, pink tongue, pale tongue, yellow-coating tongue, and white-coating tongue. This result can classify the tongue color. Therefore, it is necessary to use color features to convert the RGB color space into HSV color space while making the tongue image retrieval.

The HSV space can be obtained by the nonlinear changes of the RGB space; the conversion formula is as follows:

(3) V=max⁡R,G,B,S=V−min⁡R,G,BV.

Set

(4) r′=V−RV−min⁡R,G,B,g′=V−GV−min⁡R,G,B,b′=V−BV−minR,G,B.

Then,

(5) H′=5+b′,R=max⁡R,G,B,  G=min⁡R,G,B1−g′,R=maxR,G,B,  G≠minR,G,B1+r′,G=maxR,G,B,  B=minR,G,B3−b′,G=maxR,G,B,  B≠minR,G,B3+g′,B=maxR,G,B,  R=minR,G,B5−r′,other.H=60×H′

We can know from the above formula that

(6) H∈0,360°,S∈0,1,V∈0,1.

The main color of the image can be extracted by clustering method, and the selection of the initial cluster centers has great influence on the results of the image color classification in GLA algorithm. Because the division of the tongue image color is not obvious, the effect of the initial clustering center of the random selection is not good. In this section, we use an iterative method to determine the initial clustering number and clustering center and then use GLA algorithm to extract the main color histogram.

The specific algorithm process to determine the number of primary colors and the initial color is as follows:

(1) To specify color image *I*(*x*, *y*), set its scale as *I*(*x*, *y*); if color space is RGB space, convert it into HSV space according to formulae (3)–(5).
(2) Quantify the HSV space to nine subsections; the formula is as follows:
  (7) area=0,v≤0.2black1,s≤0.1,  0.2<v≤0.8gray2,s≤0.1,  0.9<v≤1white3+h′otherh′=0,h∈315,360∪0,20red1,h∈20,75yellow2,h∈75,155green3,h∈155,190cyan4,h∈190,260blue5,h∈260,315purple.

Scan image *I* and calculate the number of pixels belonging to the nine subspace *s*_0_, *s*_1_,…, *s*_8_ and the probability of the image *p*_0_, *p*_1_,…, *p*_8_, respectively.

(3) Set a threshold, calculate the interval of which *p*_*i*_ > *T* number, and record the value of the space area, stored in the array MC[*k*]; in the experiment, choose *T* = 15%.
(4) The number of intervals *k* determined by step (3) is the number of the main colors. And MC[*k*]'s value area is just the approximate range of the main color but cannot be used as the main color of the image. For example, MC[*k*] = 4's color represents yellow, but it can be divided into dark yellow, light yellow, and so on. Therefore, we still need to continue to iteratively calculate the main color of image.

**Figure 1:**
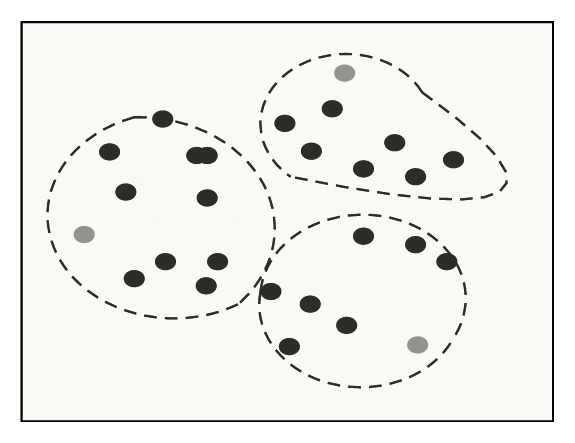
Clustering example graph of step (I).

**Figure 2:**
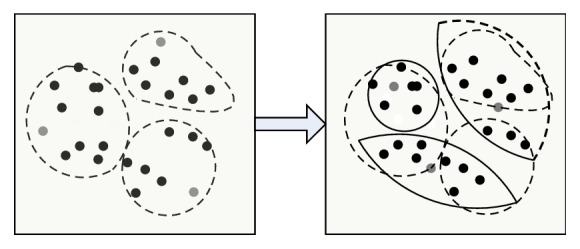
Clustering example graph of step (II).

**Figure 3:**
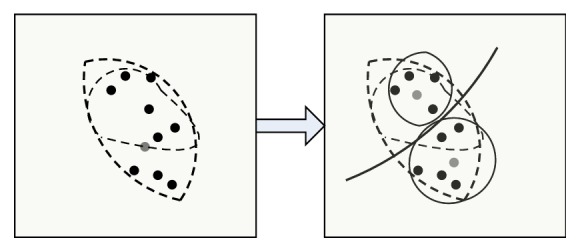
Clustering example graph of step (IV).

**Figure 4:**
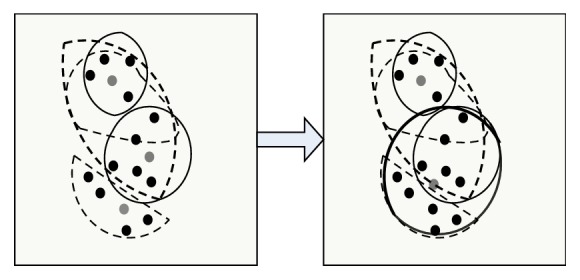
Clustering example graph of step (V).

The original main color MC[*k*] and the main color number *k* are used to obtain the main color by GLA algorithm, and then calculate the main color histogram; the steps are as follows:

(I) Classify each pixel *I*′(*j*) in the image. According to formula (8), divide *I*′(*j*) to interval where its pixel values are close to initial main color. *ω*_*i*_ is weighting coefficient.  Figure 1 describes clustering example graph of step (I).
  (8) $$\begin{array}{c} d_{i}=\sum \omega_{i}{\left\|\;I^{\prime }\left(\;j\;\right)-MC_{i}\;\right\|}^{2}. \end{array}$$
(II) Clarify cluster center. Recalculate every color interval's cluster center as new color after classifying pixels. *n*_*i*_ is the number of pixels in the MC_*i*_ interval.  Figure 2 describes clustering example graph of step (II).
  (9) $$\begin{array}{c} MC_{i}=\frac{\sum n_{i}I^{\prime }\left(j\right)}{\sum n_{i}}. \end{array}$$
(III) Repeat executed step (I) and step (II), until cluster center MC[*k*] does not change.
(IV) Perform the splitting operation. According to formula (10), calculate the errors between every color cluster interval, if error is greater than threshold *T*_1_, dividing the color interval into two new color intervals and calculating the center of new interval, MC_new1_ = MC_*i*_ − *d*/2, MC_new1_ = MC_*i*_ + *d*/2. Repeat steps (I), (II), and (III).  Figure 3 describes clustering example graph of step (IV).
  (10) $$\begin{array}{c} d_{i}=\frac{1}{n_{i}}\sum \sqrt{{\left(I^{\prime }\left(j\right)-MC_{i}\right)}^{2}}. \end{array}$$
(V) Perform merging operation. Calculate the distance of cluster centers, if the distance of two color cluster intervals is less than threshold *T*_2_, unify the two interval. According to formula (11), the new clustering center is calculated. Repeat steps (I), (II), and (III).  Figure 4 describes clustering example graph of step (V).
  (11) $$\begin{array}{c} MC_{new}=\frac{\left(n_{i}MC_{i}+n_{j}MC_{j}\right)}{n_{i}}+n_{j}. \end{array}$$
(VI) Perform the clustering end. While MC[*k*] does not change, divide or unify the cluster end.

To the *M∗N* color image *I*(*x*, *y*), use algorithm in Section 3.1 to get image main color descriptor as follows:

(12) F=c1,μ1,c2,μ2,…,ck,μk=c1,n1M∗N,c2,n2M∗N,…,ck,nkM∗N,

where *c*_*i*_ is the main color and *μ*_*i*_ presents the probability that *c*_*i*_ happen.

**Figure 5:**
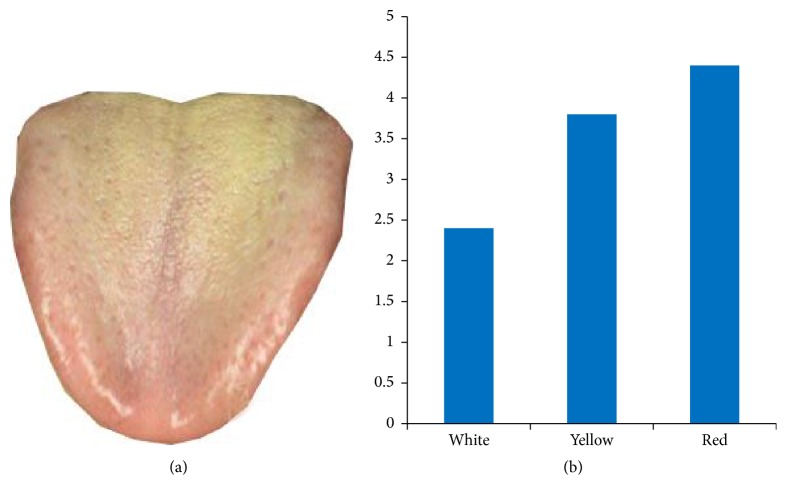
Tongue image and its main color histogram. (a) Original image. (b) Main color histogram.

Figure 5 describes a tongue image and the main color histogram extracted from the algorithm.

### 3.2. Improved Edge Histogram Extract Algorithm

The ability of color feature to distinguish tongue images containing dry fur, crack, and other prick space prominent positions is not strong. Using texture features of tongue image edge to describe the tongue image retrieval can achieve better retrieval effect on spatial distribution of tongue image. In Section 2.2, the edge histogram extraction algorithm only describes the local edge information of the image and the improved algorithm adds a global edge histogram and a semiglobal edge histogram to make up the shortage.

**Figure 6:**
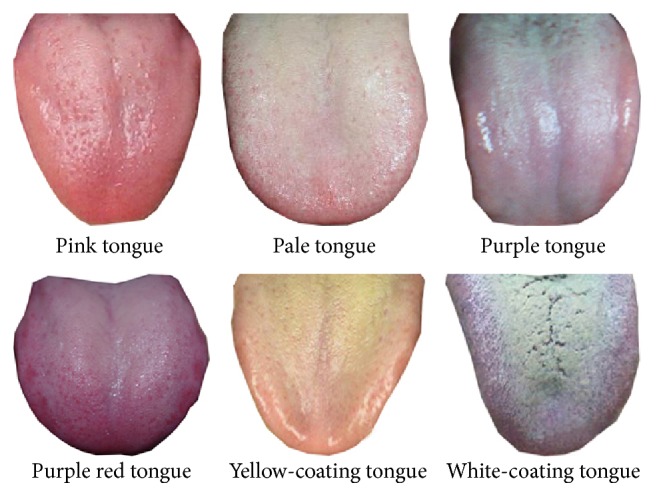
Six tongue image samples with Color Feature in database.

**Figure 7:**
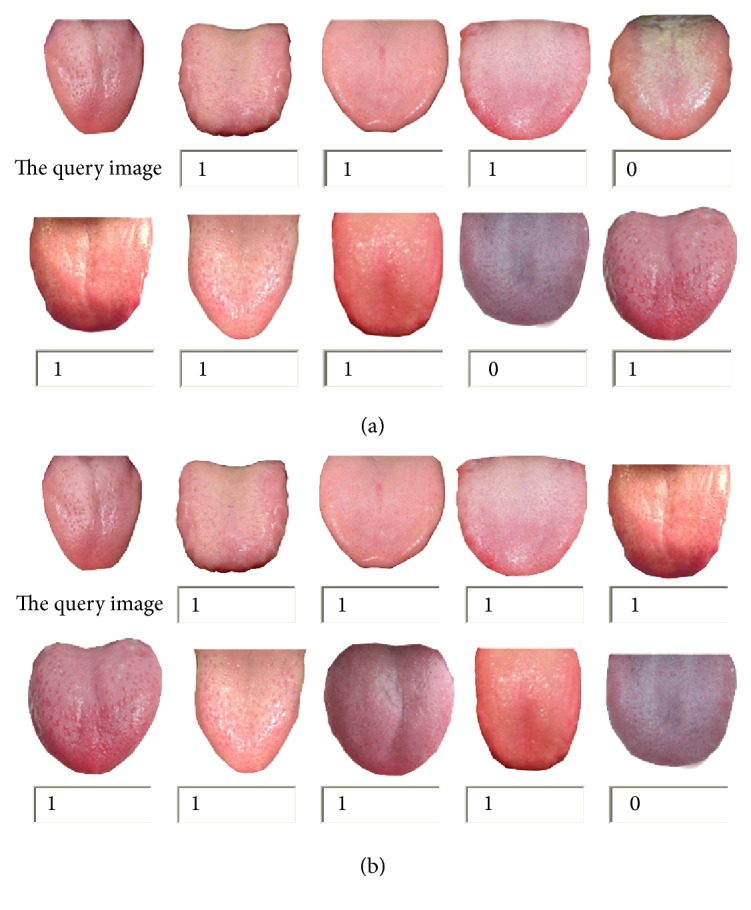
Results of two search algorithms based on color. (a) The top 9 results of tradition main color retrieval. (b) The top 9 results of improved main color histogram retrieval.

**Figure 8:**
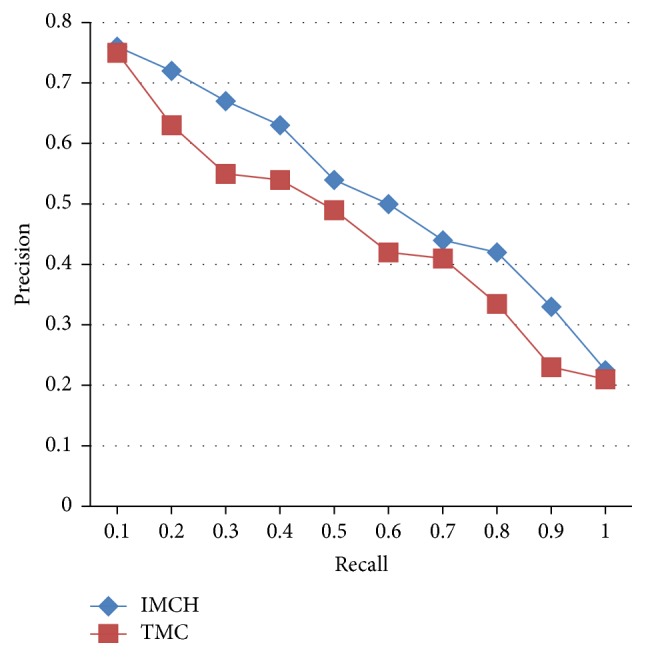
Average precision-recall curve of IMCH and TMC.

**Figure 9:**
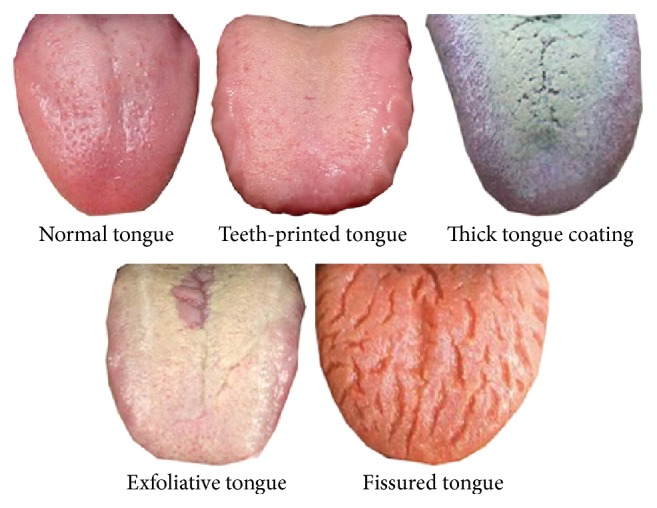
Six tongue image samples with Texture Feature in database.

**Figure 10:**
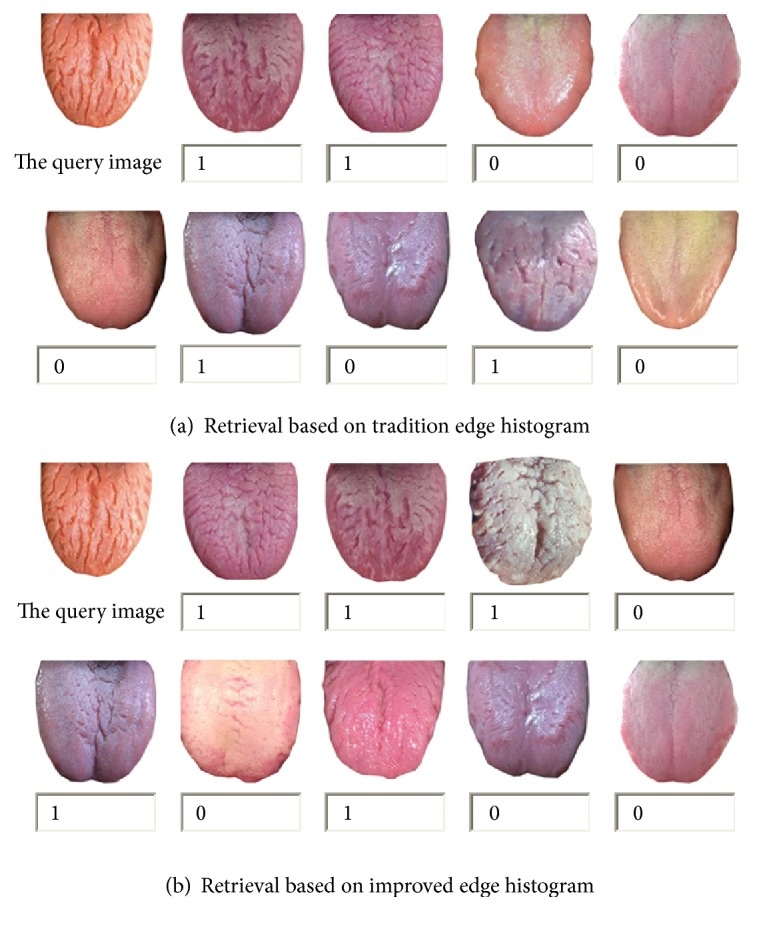
Results of two search algorithms based on texture. (a) The top 9 results of tradition edge histogram retrieval. (b) The top 9 results of improved edge histogram retrieval.

**Figure 11:**
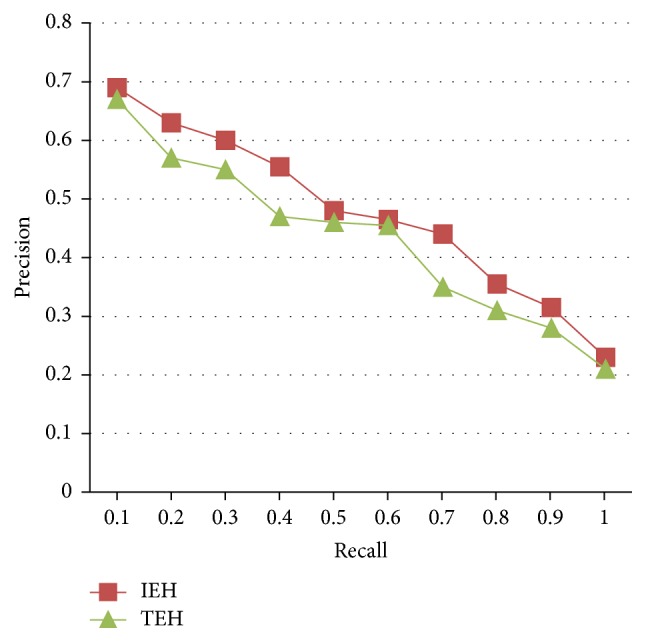
Average precision-recall curve of IEH and THE.

The improved edge histogram extract method's steps are as follows:

(1) Set *I*(*x*, *y*) to be a gray image whose scale is *M∗N*; gray level is *L*; if *I* is color image in RGB space, use the following formula to convert color image into gray image:
  (13) $$\begin{array}{c} g=0.299*r+0.587*g+0.144*b. \end{array}$$
(2) Divide *I* into 4 × 4 subimages, on average, *I*_1_,…, *I*_16_. Calculate every subimage's local edge histogram; every subimage contains 5 bin (0°, 90°, 45°, 135° and nondirection), so the whole image has 16 × 5 bin = 80 bin.
(3) Divide every subimage *I*_*i*_ into fixed number of image blocks; the area of image block changes as the area of the whole image. The number of image blocks in experiment is 256.
(4) Every subimage block can be seen as four 2 × 2 macroblocks; each of the macroblocks' edge detection operator in each direction is not the same. Calculate the five kinds of edge value of each image block and take the maximum value; if the maximum value is greater than the threshold value, then set the direction as the edge of the image block. Experimental results show that the best threshold is 20. Direction *θ*'s edge values are calculated as follows:
  (14) $$\begin{array}{c} E_{\theta }=\left|\;\overset{3}{\underset{i=0}{\sum }}a_{i}\left(\;m,n\;\right)*f_{\theta }\left(\;i\;\right)\;\right|, \end{array}$$
where *a*_*i*_(*m*, *n*) represents the average gray value of the *i* macroblock and *f*_*θ*_(*i*) represents *i* macroblocks' edge detection operator in direction *θ*. *θ* is 0°, 90°, 45°, 135°, and no direction.
(5) Get the subimage *I*_*i*_'s 5 bin edge histogram from 256 image blocks; the whole image of the edge histogram is 80 bin.
(6) Normalize and quantify the edge histogram got in step (5), and then with nonlinear quantified value 80 bin which has to be normalized, each histogram uses fixed 3 bit to encode reduced amount of computation.
(7) Global histogram represents the edge distribution information of whole image, calculated by adding and averaging the distribution information of the subimage in five directions; the dimension of the global histogram is 5. Set the local edge histogram of the image as EH; then the global histogram is
  (15) $$\begin{array}{c} GH_{\theta }=\overset{16}{\underset{i=1}{\sum }}EH_{\theta }\left(\;i\;\right). \end{array}$$
(8) Semiglobal histograms represent image region horizontal, vertical, and adjacent block edge information. As shown in Figures 3–11, 1~4 subblocks represent the vertical edge information, 8 subblocks represent the horizontal edge information, 9–13 subblocks represent adjacent block edge information, and semiglobal histogram of the whole image is 13 × 5 bin = 65 bin.

### 3.3. Feature Extract

To the *M∗N* color image *I*(*x*, *y*), use the algorithm in Section 3.1 to get image main color descriptor as follows:

(16) F=c1,μ1,c2,μ2,…,ck,μk=c1,n1M∗N,c2,n2M∗N,…,ck,nkM∗N,

where *c*_*i*_ is the main color and *μ*_*i*_ represents the probability that *c*_*i*_ happen.

Get the image's local edge histogram by using algorithm in Section 3.2  EH = {*E*_0°_, *E*_45°_, *E*_90°_, *E*_135°_, *E*_non-direction_}; global histogram GH = {*g*_1_, *g*_2_,…, *g*_5_} and semiglobal histogram SGH = {*S*_0°_, *S*_45°_, *S*_90°_, *S*_135°_, *S*_non-direction_}.

Consider *E*_*θ*_ = {*e*_*θ*,1_, *e*_*θ*,2_,…, *e*_*θ*,16_}, where *e*_*θ*,*j*_ represents the image edge value in *j* subimage of *θ* direction: *g*_*i*_ = ∑_*j*=1_^16^*e*_*θ*_*i*_,*j*_, *θ*_1_ = 0°, *θ*_2_ = 45°, *θ*_3_ = 90°, *θ*_4_ = 135°, *θ*_5_ = nondirection; *S*_*θ*_ = {*s*_*θ*,1_, *s*_*θ*,2_,…, *s*_*θ*,13_}, *s*_*θ*,1_ represents 13 subblocks standing for semiglobal information of image's edge value in *θ* direction.

### 3.4. Similarity Measurement

To a given image, the algorithm can extract the main color *F*, the local edge histogram EH, the global edge histogram GH, and the semiglobal edge histogram SGH, where EH is an 80-dimensional vector and SGH's dimension is 65.

Set the histograms EH_*q*_, GH_*q*_, SGH_*q*_ and EH_*t*_, GH_*t*_, SGH_*t*_ as *Q* and *T*'s local edge histogram, global edge histogram, and semiglobal edge histogram. Adding the weight of the global histogram to increase the impact of the image, *Q* and *T*'s texture similarity is defined as

(17) DQ,T=∑i=079EHqi−EHti+5×∑i=04GHqi−GHti+∑i=064SGHqi−SGHti.

The proposed algorithm is a comprehensive retrieval for the color and texture of the image. If the distance between query image *Q*'s main color histogram and target image *T*'s main color histogram is *d*_1_, edge histogram gets the distance *d*_2_, *d*_1_'s range is [0, max_1_], and *d*_1_'s range is [0, max_2_]. The greater the distance value is, the more the two images are not similar. To make *d*_1_ and *d*_2_ able to be compared, normalize them: *d*_1_ = (max_1_ − *d*_1_)/max_1_; *d*_2_ = (max_2_ − *d*_2_)/max_2_.

*d*_1_ and *d*_2_'s range after normalization is [0,1]. If two images are the most similar, the similarity measurement is 1; otherwise, the least similarity measurement is 0 and the similarity measurement is a value of 0~1. While *d* = *ω*_1_*d*_1_ + *ω*_2_*d*_2_ retrieves the main color and edge histogram, using the distance of similarity, *ω*_1_ represents the weight of main color and *ω*_2_ represents the weight of texture feature. Generally speaking, the weight is 0.6 : 0.4. To different image information and practical applications, we can increase a certain weight to achieve better retrieval results.

## 4. Experiment Result and Analysis

### 4.1. Color Feature Retrieval Experiment

To evaluate the performance of Section 3.1 of the proposed algorithm, in Experiment  1, we used 268 tongue images as experimental samples; these images are divided into several colors in advance by the doctor. The total tongue images were divided into 6 categories, respectively, pink tongue, pale tongue, purple tongue, purple red tongue, yellow-coating tongue, and white-coating tongue, and each category contains at least 30 images. Six tongue image samples are shown in Figure 6.

We randomly selected a sample from each category as an example of tongue image and then retrieved it in the database. System first calculated the color feature vector and then similarity matched the color feature vector of the tongue image in the feature library. The similarity of the Euclidean distance is used in the paper, finally the returned tongue image is most similar to the sample.

Take pink tongue as an example, Figure 7 represents the first nine images based on the traditional main color algorithm and the improved algorithm of the main color.

Two kinds of algorithm's retrieval performance can be displayed from the retrieval system. In Figure 3, each set of images' upper left corner image is the image to be retrieved; the others are retrieval results. “1” is the related image and “0” is not related image. From experimental results, we can see that the improved main color histogram algorithm can usually be compared with the most similar images of those related images in advance, which is more consistent with the human visual perception.

To further compare the performance of the two algorithms, we, respectively, used traditional principal color algorithm and improved main color histogram algorithm to make a lot of tongue image retrieval experiments, calculated average precision of two algorithms in different tongue images, and then calculated the average precision of each algorithm to compare two algorithms' integrated retrieval performances. Precision is defined as the ratio between the target images and the all images searched: precision = *M*/*L*, and recall is defined as the ratio between the target images in the result queue and the target images in the database: recall = *M*/*D*. Here *L* represents the total number of images returned by retrieval results, *M* represents the number of target images associated with the query image in the query results, and *D* represents the number of target images from image library, related to the image to be queried. The higher the precision is, the better the algorithm retrieval becomes.

Six groups of images are selected from tongue image database to build a retrieval set, forming 12 times retrieval.  Figure 4 shows the precision comparing results between this paper's algorithm and tradition main color retrieval method. TMC represents the retrieval results based on tradition main color retrieval method, and IMCH represents the retrieval results based on the improved main color histogram method.

Experimental results show that the improved main color histogram algorithm is better than the traditional main color histogram in the search results. Although differences of the statistical results are small, we can obviously feel the effect of the retrieval greatly improved. At the same time, the position of the relevant tongue is also more forward and focused.

### 4.2. Texture Feature Retrieval Experiment

To evaluate the performance of Section 3.2 algorithms, experiment used the same tongue images ditto, which are divided into 5 categories: normal tongue, teeth-printed tongue, thick tongue coating, exfoliative tongue, and fissured tongue, and each category contains at least 30 images. Five tongue image samples are shown in Figure 9.

We randomly selected a sample from each category as an example of tongue image and then retrieved it in the database. The similarity of the Euclidean distance is used in this paper, and finally the returned tongue image is most similar to the sample.

Using fissured tongue image as an example, Figure 10 represents the retrieval results according to traditional edge histogram algorithm and improved edge histogram algorithm, the first nine images sorted according to the size of the similarity.

We repeatedly retrieved each type of tongue image.  Figure 11 shows the precision and recall ratio comparing results between this paper's algorithm and tradition edge histogram retrieval method. TEH represents the retrieval results based on tradition edge histogram retrieval method, and IEH represents the retrieval results based on the improved edge histogram method.

Experimental results show that improved edge histogram's retrieval precision and recall ratio is slightly higher than that of the traditional edge histogram. Although differences of the statistical results are small, we can obviously feel the effect of the retrieval greatly improved. At the same time, the position of the relevant tongue is also more forward and focused.

### 4.3. Comprehensive Feature Retrieval Experiment

To analyze the function of comprehensive color and texture feature in tongue image retrieval, we select a set of tongue images which have prominent color and texture features for the experiment and then randomly select a tongue image as the query image. In order to achieve better retrieval results, the weight of color and texture features is set to 0.6 and 0.4, respectively.

**Figure 12:**
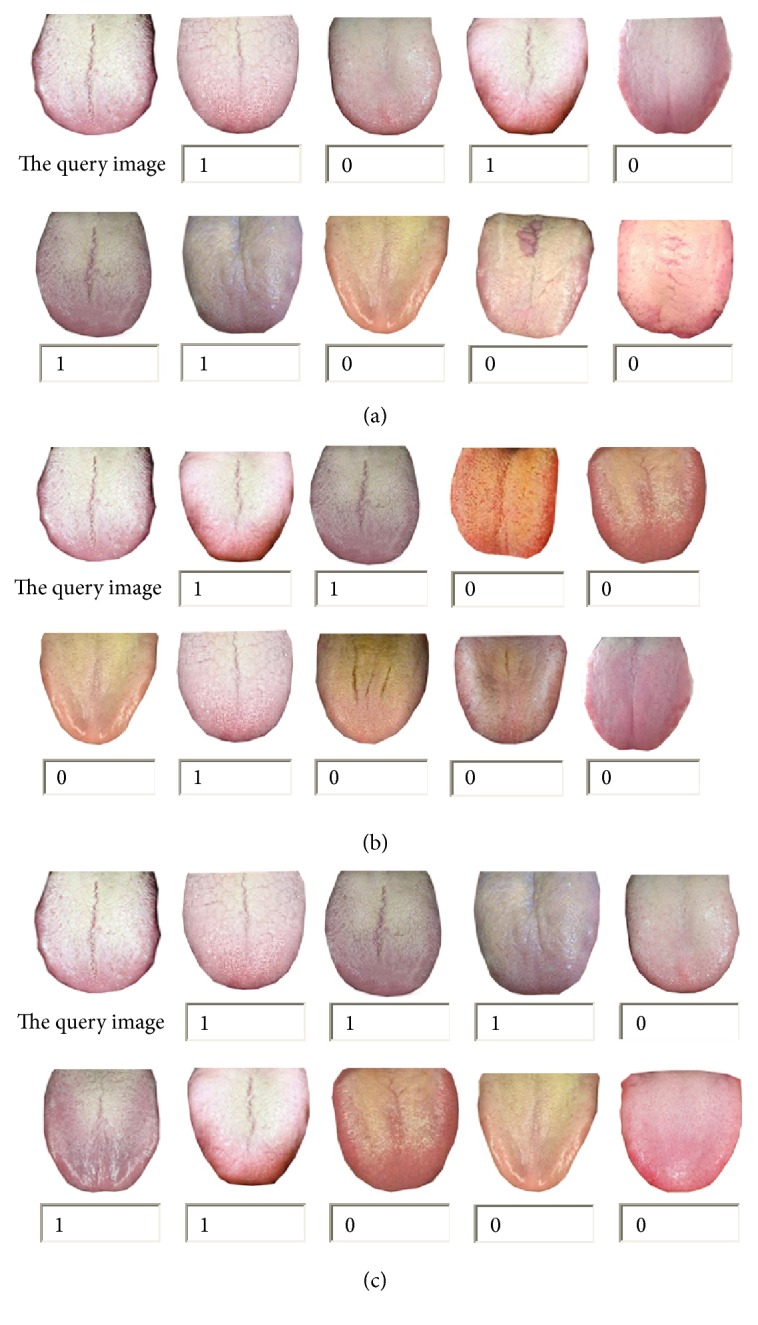
Results of three search algorithms based on color and texture. (a) The top 9 results of improved main color histogram retrieval. (b) The top 9 results of improved edge histogram retrieval. (c) The top 9 results of comprehensive main color and edge histogram retrieval.

Figure 12 represents the retrieval results of the main color algorithm, improved edge histogram algorithm, and image retrieval algorithm based on the main color and edge histogram, in accordance with the first nine images according to the size of the similarity.

Figure 12(a) is the result of only using color features. The similarity is gradually reduced from left to right, from top to bottom. Although the retrieval tongue image is similar to the query image in the color, the texture pattern of the last two images is obviously different from the query image.  Figure 12(b) is the result of only using texture features. Although the retrieval performance is better than that in Figure 12(a), but it retrieved a tongue image with entirely different color.  Figure 12(c) shows a comprehensive color and texture of the two features and for the same tongue image retrieval results; we can see that the result is more accurate than the single feature search results.

To further compare the performances of the three algorithms, the paper uses a training set method, with 5 times cross validation, and the distribution feature of the training set is sufficient to describe the distribution feature of the entire image set. In this way, when adding new image to the training set, it will not affect the distribution feature of the entire image database and each image in training set is used in experiment as a query image. Calculate the average precision and recall ratio of the training set of images; the experimental results are shown in Table 1.

From Table 1, we can see that when using algorithm 1 to retrieve tongue images, precision effect is slightly higher than that of algorithm 2; the precision of algorithm 3 tongue image retrieval is higher than that of the previous two algorithms, but the average recall ratio is low. The reason is the difference of the tongue images' color performance, which can be expressed better by the feature extraction of algorithm 1. Because the difference of tongue image texture feature is not such obvious, the extraction of the feature of algorithm 2 is difficult. Algorithm  3 can focus on both color and texture features, so it achieves a higher precision and recall ratio.

A large number of tongue image retrieval experiments show that, due to the great difference of the tongue images, using color or texture features in retrieval would be better for some tongue images. Therefore, to know tongue images in the practical application, we first judge the color and texture of the tongue image according to human's vision and then select the different retrieval methods and weights to obtain more satisfactory results.

## 5. Conclusion

The paper takes tongue image as an example; the research focuses on the key technology of image feature extraction and the technology research of the last layer which is the measurement of the similarity distance, so as to realize the content-based retrieval of the tongue image with a specific diagnostic value.

This paper first uses the iterative method to extract the initial main colors and the number of them and then gets the main color histogram by GLA algorithm. Considering the deficiency of expressing tongue image information by using color feature, the paper analyzes tongue image further and puts an algorithm to get tongue image's texture feature. The new method combines the improved main color histogram descriptor and edge histogram descriptor to give different weights to the comprehensive retrieval. Experiments on the 268 images including normal tongue, teeth-printed tongue, thick tongue coating, exfoliative tongue, fissured tongue, and variety of colors verify the effectiveness and robustness of this method. Experiments show that new method can improve the detection rate of lesion in tongue images.

The content-based image retrieval technology has a certain practical significance in the Traditional Chinese Medicine. Using this technique, the information can be extracted directly from the tongue image database, which avoids the subjectivity of the manual annotation of the tongue image and greatly reduces the manual workload. The research according to this subject will have broad application prospects. The results of objectivity tongue diagnosis play a positive role in promoting the Chinese traditional medicine research. How to combine with clinicians stagnant standard to extract more high level feature and identify the lesion images aiming at the different manifestations of the disease is a subject that needs further research.

## Figures and Tables

**Algorithm 1:**
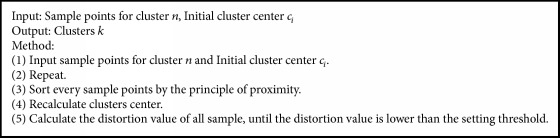
GLA clustering algorithm (GLA algorithm).

**Table 1:** Search result analysis.

Method	Precision	Recall
Improved main color histogram	0.4036	0.3481
Improved edge histogram	0.3983	0.2953
Comprehensive main color and edge histogram	0.5113	0.3620
